# *Synchrospora* gen. nov., a New Peronosporaceae Genus with Aerial Lifestyle from a Natural Cloud Forest in Panama

**DOI:** 10.3390/jof9050517

**Published:** 2023-04-27

**Authors:** Thomas Jung, Yilmaz Balci, Kirk D. Broders, Ivan Milenković, Josef Janoušek, Tomáš Kudláček, Biljana Đorđević, Marilia Horta Jung

**Affiliations:** 1 Phytophthora Research Centre, Faculty of Forestry and Wood Technology, Mendel University in Brno, 613 00 Brno, Czech Republic; ivan.milenkovic@mendelu.cz (I.M.); josef.janousek@mendelu.cz (J.J.); tomas.kudlacek@mendelu.cz (T.K.); biljana.dordevic@mendelu.cz (B.Đ.); marilia.jung@mendelu.cz (M.H.J.); 2 Phytophthora Research and Consultancy, 83131 Nußdorf, Germany; 3 USDA-APHIS Plant Protection and Quarantine, 4700 River Road, Riverdale, MD 20737, USA; yilmaz.balci@usda.gov; 4 Smithsonian Tropical Research Institute, Apartado Panamá, Panama City 0843-03092, Panama; kirk.broders@usda.gov; 5 USDA, Agricultural Research Service, National Center for Agricultural Utilization Research, Mycotoxin Prevention and Applied Microbiology Research Unit, Peoria, IL 61604, USA; 6 Faculty of Forestry, University of Belgrade, Kneza Višeslava 1, 11030 Belgrade, Serbia

**Keywords:** oomycete, evolution, phylogeny, caducity, synchronous sporulation, adaptation, homothallic, tropical, canopy, leaf pathogen

## Abstract

During a survey of *Phytophthora* diversity in Panama, fast-growing oomycete isolates were obtained from naturally fallen leaves of an unidentified tree species in a tropical cloud forest. Phylogenetic analyses of sequences from the nuclear ITS, LSU and *ßtub* loci and the mitochondrial *cox1* and *cox2* genes revealed that they belong to a new species of a new genus, officially described here as *Synchrospora* gen. nov., which resided as a basal genus within the *Peronosporaceae*. The type species *S. medusiformis* has unique morphological characteristics. The sporangiophores show determinate growth, multifurcating at the end, forming a stunted, candelabra-like apex from which multiple (8 to >100) long, curved pedicels are growing simultaneously in a medusa-like way. The caducous papillate sporangia mature and are shed synchronously. The breeding system is homothallic, hence more inbreeding than outcrossing, with smooth-walled oogonia, plerotic oospores and paragynous antheridia. Optimum and maximum temperatures for growth are 22.5 and 25–27.5 °C, consistent with its natural cloud forest habitat. It is concluded that *S. medusiformis* as adapted to a lifestyle as a canopy-dwelling leaf pathogen in tropical cloud forests. More oomycete explorations in the canopies of tropical rainforests and cloud forests are needed to elucidate the diversity, host associations and ecological roles of oomycetes and, in particular, *S. medusiformis* and possibly other *Synchrospora* taxa in this as yet under-explored habitat.

## 1. Introduction

The Peronosporaceae, a sister family of the Pythiaceae, belong to the Peronosporales, Peronosporomycetes, Stramenipila and currently comprise 25 genera, i.e., *Calycofera*, *Halophytophthora*, *Nothophytophthora*, *Phytophthora*, *Phytopythium* and 20 genera of downy mildews (DMs) [1,2,3,4,5,6,7,8,9]. Several phylogenetic studies have demonstrated that *Phytophthora* is monophyletic, with all DMs residing as two separate clades within *Phytophthora*, rendering *Phytophthora* paraphyletic with respect to its DM descendants [4,6,7,8,10,11,12,13,14,15,16]. Due to the monophyly and the biological and structural cohesion of *Phytophthora*, its historic and social impacts and its importance in scientific communication and biosecurity protocols, Brasier et al. [16] proposed to conserve the current nomenclatural status. They also argued that paraphyletic jumps, such as the emergence of descendants with a new lifestyle (the DMs) from an ancestral and evolutionary successful, cohesive genus (*Phytophthora*), should be considered a normal feature of evolution. Unlike *Phytophthora*, the genus *Pythium* was, in multigene phylogenetic analyses, shown to be polyphyletic [10,12,17,18,19] and, consequently, divided into *Pythium sensu stricto* and four new genera, i.e., *Phytopythium* (syn. *Ovatisporangium*), *Elongisporangium*, *Globisporangium* and *Pilasporangium* [20,21,22,23]. Because of its phylogenetic relatedness and morphological similarity to *Phytophthora, Phytopythium* was assigned to the Peronosporaceae, whereas the other four genera constitute the Pythiaceae [4,6,9,22].

In 1952, Gäumann [24], based on morphological and pathogenic data, postulated an evolutionary development from the saprophytic *Pythium* species via hemibiotrophic or necrotrophic *Phytophthora* species to the obligate biotrophic downy mildews, which was confirmed later by various phylogenetic analyses. While *Calycofera*, *Halophytophthora* and *Phytopythium* species are mostly saprotrophs and/or necrotrophic opportunistic plant pathogens, most *Phytophthora* species have a hemibiotrophic or necrotrophic lifestyle as primary plant pathogens. In addition, predominantly aquatic *Phytophthora* species within phylogenetic clades 6, 9 and 10 have a partially saprotrophic lifestyle, and 46.7% of the 210 known culturable *Phytophthora* species are able to thrive as saprotrophs in waterbodies [5,9,16,25,26,27,28,29,30,31,32,33,34]. In contrast, all ca 900 DM species are highly specialised, obligate biotrophic plant pathogens [4,13,14,35]. The genera *Calycofera*, *Halophytophthora* and *Phytopythium* lack sporangial caducity congruent with their soilborne and/or aquatic lifestyle. In contrast, 75% of the eight described *Nothophytophthora* species, 26.7% of the 210 described *Phytophthora* species and all ca 900 DM species have caducous sporangia (or conidia), enabling an aerial or partially aerial lifestyle [4,5,6,9,13,14,16,22,31,34,35,36].

Since the 1960s, the number of devastating epidemics caused by introduced invasive *Phytophthora* species, including *P. austrocedrae*, *P. cinnamomi*, *P. lateralis*, *P. plurivora*, *P. ramorum*, *P.* ×*alni* or *P.* ×*cambivora* in both managed and natural ecosystems, has been increasing exponentially [16,26,37,38,39,40,41,42,43,44,45,46,47,48,49,50,51,52,53,54,55,56,57,58,59,60,61,62,63,64,65,66,67,68,69,70,71]. This has stimulated extensive *Phytophthora* surveys in previously unexplored natural ecosystems across most continents using classical baiting and isolation methods and sometimes metagenomic approaches as well. These surveys have unveiled an unprecedented diversity of described and previously unknown *Phytophthora* taxa in various ecological niches [6,16,30,31,41,44,45,47,72,73,74,75,76,77,78,79,80,81,82,83,84,85,86,87,88,89,90,91,92,93,94].

During a survey of *Phytophthora* diversity in Central America, fast-growing isolates that morphologically resemble the *Phytophthora* species were obtained from naturally fallen leaves in a tropical cloud forest in Panama. A preliminary phylogenetic analysis of ITS rDNA sequences suggested that they belong to a previously unknown distinct species from a potentially new Peronosporaceae genus. In this study, morphological and physiological characteristics were used in combination with DNA sequence data from three nuclear, i.e., ITS, part of the 28S large subunit (LSU) and β–tubulin, and the two mitochondrial *cox*1 and *cox2* gene regions to characterise and officially describe the new oomycete genus as *Synchrospora* gen. nov. and the new taxon as *S. medusiformis* sp. nov.

## 2. Materials and Methods

### 2.1. Isolate Collection and Maintenance

Details of all isolates used in the phylogenetic, morphological and temperature–growth studies are given in Table 1 and Table S1. Sampling and isolation methods from naturally fallen necrotic leaves were according to [45]. For all isolates, single hyphal tip cultures were produced under the stereomicroscope from the margins of fresh cultures on V8–juice agar (V8A; 20 g agar, 3 g CaCO_3_, 100 mL Campbell’s V8 juice, 900 mL distilled water). Stock cultures were maintained on V8A and carrot agar (CA; 20 g agar, 3 g CaCO_3_, 200 g carrots, 1000 mL distilled water; [68]) at 20 °C in the dark. All isolates of the new genus *Synchrospora* were preserved in the culture collection maintained at the Phytophthora Research Centre, Mendel University, in Brno. The ex–type culture was deposited at the Westerdijk Fungal Biodiversity Institute, Utrecht, Netherlands (Table 1), and the novel taxonomic description and nomenclature were submitted to MycoBank (www.mycobank.org).

### 2.2. DNA Extraction, Amplification and Sequencing

For all five *Synchrospora* isolates obtained in this study, 10 ex-type isolates from the genera *Halophytophthora* and *Nothophytophthora* and the neotype isolates of *P. infestans* and *P.* ×*cambivora* DNA were extracted from c. 15–100 mg of mycelium scraped from 1–3-wk-old V8A cultures, placed into 2 mL homogenisation tubes (Lysis Matrix A; MP Biomedicals, Irvine, USA) and disrupted using a Precellys Evolution instrument (Bertin Technologies, Montigny-le-Bretonneux, France) until the mixture was homogenous. DNA was purified using the Monarch Genomic DNA Purification Kit (New England Biolabs, Ipswich, USA) and treated with RNase A following the manufacturer’s protocol for tissue samples. DNA was eluted with 100 μL of pre-warmed elution buffer and preserved at −80 °C for long-term storage. Three nuclear gene regions, i.e., the internal transcribed spacer region (ITS1–5.8S–ITS2) of the ribosomal RNA gene (ITS), the 5′ terminal domain of the large subunit (28S-LSU) of the nuclear ribosomal RNA and β-tubulin (*βtub*), and the two mitochondrial genes cytochrome-c oxidase 1 (*cox1*) and 2 (*cox2*), were amplified and sequenced (Table 2). PCR amplifications were performed using a LightCycler 480 II instrument (Roche, Basel, Switzerland) or Eppendorf Mastercycler nexus GSX1 (Eppendorf, Hamburg, Germany). All primers were synthesised by Elizabeth Pharmacon spol. s.r.o. (Brno, Czech Republic). Their annealing temperatures were estimated using a Tm calculator (http://tmcalculator.neb.com/#!/main, accessed on 24 February 2023) and adjusted empirically, according to observed PCR amplification rates. Table 2 provides a comprehensive overview of the PCR conditions and primers used. Primer FM35_Oom2 was designed using a global alignment of *cox2* sequences from all described species of *Calycofera*, *Halophytophthora*, *Nothophytophthora*, *Phytophthora*, *Phytopythium* and selected species from other oomycete genera. Each nucleotide was carefully checked to identify whether it is conserved and, if necessary, replaced by a degenerate nucleotide. PCR products were visualised by gel electrophoresis (300 V; 5 min) using 2% agarose gel stained by DNA Stain G (SERVA, Heidelberg, Germany). All amplicons were purified and sequenced in both directions by Eurofins Genomics GmbH (Cologne and Ebersberg, Germany) using the amplification primers, except for the LSU amplicons, which required two additional primers (Table 2). Electropherograms were quality checked, and forward and reverse reads were compiled using Geneious Prime^®^ v. 2022.0.2 (Biomatters Ltd., Auckland, New Zealand). Pronounced double peaks were considered as heterozygous positions and labelled according to the IUPAC (International Union of Pure and Applied Chemistry; https://iupac.org, accessed on 24 February 2023) coding system. All sequences generated in this study were deposited in GenBank, and accession numbers are given in Table S1.

### 2.3. Phylogenetic Analyses

The sequences obtained in this work were complemented with sequences deposited in GenBank. Sequences of all loci used in the analyses were aligned using the MAFFT v. 7 [95] plugin within the Geneious Prime^®^ v. 2023.0.4 software (Biomatters Ltd., Auckland, New Zealand) by the E-INS-I strategy (ITS) or the G-INS-I strategy (all other loci).

To study the phylogenetic position of the potentially new genus among other oomycete genera, a five-partition (LSU–ITS–*βtub*–*cox1*–*cox2*) dataset of representative species from all genera of the Peronosporaceae and Pythiaceae together with five isolates from the new species were analysed with *Saprolegnia parasitica* (CBS 223.65) and *Aphanomyces euteiches* (ATCC 201684) as outgroups (dataset: 60 isolates and 4954 characters). Maximum likelihood (ML) and Bayesian (BI) analyses were carried out.

Within the ML analysis, each gene was treated as a separate partition, and best-fitting evolutionary models were estimated with PartitionFinder 2 [96] based on the corrected Akaike Information Criterion (AICc). The analysis was done for both linked and unlinked branch lengths, and the results were the same. The largest set of models possible was tested, including models with base frequencies estimated using maximum likelihood (84 models in total; option models = allx;). All possible partitioning schemes were analysed (option search = all;).

Phylogenetic inference estimated based on the ML method was produced in RAxML-NG 1.1.0 [97]. The necessary number of bootstrap replicates was determined automatically using the MRE-based bootstrapping test [98] with the default cut-off value 0.03 (option --bs-cutoff 0.03). The analysis converged before reaching the maximum number of replicates, which was set to 10,000 (option –autoMRE{10000}). As a support metric, we used Transfer Bootstrap Expectation [99]. As a summarising tree, the 50% majority rule consensus tree was created using SumTrees 4.4.0 within the Python library DendroPy 4.4.0 [100]. Edge lengths of the summarising tree were calculated as mean lengths for the corresponding edges in the input set of trees.

For the BI analysis, a Metropolis-coupled MCMC method (MC3) implemented in the CoupledMCMC package [101] with four chains (three heated and one cold) was used. The chain length was set to 20,000,000, and every 5000th state was sampled. Target switch probability was set to the recommended value of 0.234 [102,103]. Site models for individual genes were selected automatically by model averaging implemented in the bModelTest package [104]. The uncorrelated lognormal relaxed molecular clock model [105] was used. Substitutions per site were used as the unit of branch lengths of the sampled trees. Parameter estimates were summarised with TreeAnnotator 2.6.0 (part of BEAST 2) and mapped onto the 50% majority-rule consensus tree built with SumTrees 4.4.0 [100]. The option “force-rooted“ was used, telling SumTrees to treat the tree as rooted. Edge lengths were calculated as mean lengths for the corresponding edges in the input set of trees. The posterior estimates of the parameters were summarised with Tracer 1.7.1 [106]. The quality of the parameter estimates was assessed based on visual analysis of trace plots and ESS values. Proper sampling was indicated by the minimum ESS value of 200 (standard approach). Likelihood and most of the other parameters of the final tree were higher than 200. Burn-in was set to 25%. Branches with a support value ≤ 0.5 were collapsed using TreeGraph 2 [107].

Phylogenetic trees were visualised in TreeGraph2 v. 2.15.0-887 beta [107] and/or MEGA 11 v. 11.0.11 [108] and edited in figure editor programs. The dataset and trees deriving from BI and ML analyses are available from the Dryad Digital Repository (https://datadryad.org; https://doi.org/10.5061/dryad.p2ngf1vvt).

**Table 2:** PCR conditions and details of primers used for amplification and sequencing of oomycete isolates.

Locus	Primer Names	Primer Sequences (5′-3′)	Orientation	Annealing Temperature (°C); Extension Time (s)	Reference for Primer Sequences
*βtub* ^1,2^	TUBUF2 TUBUR1	CGGTAACAACTGGGCCAAGG CCTGGTACTGCTGGTACTCAG	Forward Reverse	68; 12	[12]
	Btub_F1A	GCCAAGTTCTGGGARGTSAT	Forward	66; 15	[109]
	Btub_R1A	CCTGGTACTGCTGGTAYTCMGA	Reverse		
*cox1*	OomCoxI-Levup ^1^OomCoxI-Levlo ^1^	TCAWCWMGATGGCTTTTTTCAAC CYTCHGGRTGWCCRAAAAACCAAA	Forward Reverse	60; 10	[110]
	COXF4N ^3^COXR4N ^3^	GTATTTCTTCTTTATTAGGTGC CGTGAACTAATGTTACATATAC	Forward Reverse	50; 65	[12]
*cox2*	FM35 ^1,4^ OomCoxI-Levlo	CAGAACCTTGGCAATTAGG CYTCHGGRTGWCCRAAAAACCAAA	Forward Reverse	60; 20	[110,111]
	FM35_Oom2 ^3,4^ OomCoxI-Levlo	SCNKWACCTTGGCAAWTRGG CYTCHGGRTGWCCRAAAAACCAAA	Forward Reverse	50; 80	[this study] [110]
*cox1* and *cox2* ^3,4^	FM35 COXR4N OomCoxI-Levlo ^5^ FM83_Oom ^5^ FM80_RC ^5^ COX2-R ^5^	CAGAACCTTGGCAATTAGG CGTGAACTAATGTTACATATAC CYTCHGGRTGWCCRAAAAACCAAA CHCCNATAAARAATAACCARAARTG TTTCAACAAATCATAAAGATAT CCATGATTAATACCACAAATTTCACTAC	Forward Reverse Reverse Reverse Forward Reverse	50; 150	[12,31,110,111,112,113]
ITS ^1^	ITS1 ITS4 ^6^ ITS6 ^6^	TCCGTAGGTGAACCTGCGG TCCTCCGCTTATTGATATGC GAAGGTGAAGTCGTAACAAGG	Forward Reverse Forward	63–65; 12	[10,114]
LSU ^1,7^	CTB6 LR3 ^5^ LR3R ^5^ LR7	GCATATCAATAAGCGGAGG CCGTGTTTCAAGACGGG GTCTTGAAACACGGACC TACTACCACCAAGATCT	Forward Reverse Forward Reverse	53; 20	[115,116]

^1^ PCR protocol 1: 20 µL volume containing 10.4 µL H_2_O, 4 µL Q5 Reaction Buffer (5X), 1 µL of each primer (10 μM), 0.4 µL deoxynucleotide (dNTP) mixture (Meridian Bioscience, Memphis, USA) (2.5 mM each), 0.2 µL of Q5 High-Fidelity DNA Polymerase (2 U/μL) (New England Biolabs, Ipswich, USA), and 3 µL of gDNA. Initial denaturation for 30 s at 98 °C; 35 cycles consisting of 5 s at 98 °C, 20 s at optimised annealing temperature for each primer set, optimised length of extension at 72 °C; 2 min at 72 °C for final extension. ^2^ Two primer pairs were used separately: TUBUF2/TUBUR1 or Btub_F1A/Btub_R1A. ^3^ PCR protocol 2: 20 µL volume containing 10 µL H_2_O, 4 µL PrimeSTAR GXL Buffer (5X), 0.8 µL of each primer, 1.6 µL dNTP mixture, 0.4 µL PrimeSTAR GXL DNA Polymerase (1.25 U/μL) (TaKaRa Bio, Kusatsu, Shiga, Japan), and 3 µL of gDNA. Initial denaturation for 5 s at 98 °C; 35 cycles consisting of 10 s at 98 °C, 15 s at optimised annealing temperature, optimised length of extension at 68 °C; 5 min at 68 °C for final extension. ^4^ Three different primer pairs were used separately for COX II amplification: FM35/OomCoxI-Levlo or FM35_Oom2/OomCoxI-Levlo or FM35_Oom2/COX4RN (in this case also COX I was amplified too). ^5^ Primers used exclusively for sequencing. ^6^ Two primer combinations were used separately: ITS1/ITS4 or ITS6/ITS4. ^7^ Double concentration of Q5 polymerase.

### 2.4. Morphology of Asexual and Sexual Structures

Formation of sporangia was induced by submersing two 12–15 mm square discs cut from the growing edge of a 2–4-d-old V8A colony in a 90-mm-diameter Petri dish in non–sterile soil extract (50 g of oak forest soil in 1 000 mL of distilled water, filtered after 24 h) [39]. The Petri dishes were incubated at 20 ºC, and natural daylight and the soil extract changed after ca 6 h [74]. Shape, type of apex, caducity, pedicels and special features of sporangia were recorded after 24–48 h. For each isolate, 50 sporangia were measured at ×400 using a compound microscope (Zeiss Imager.Z2), a digital camera (Zeiss Axiocam ICc3) and biometric software (Zeiss ZEN).

The formation of gametangia (oogonia and antheridia) and their characteristic features were examined after 21–30 d growth at 20 °C in the dark on V8A. For each isolate, 50 oogonia, oospores and antheridia chosen at random were measured under a compound microscope at ×400. The oospore wall index was calculated according to [117].

### 2.5. Colony Morphology, Growth Rates and Cardinal Temperatures

Colony growth patterns of all five isolates of *S. medusiformis* were described from 7-d-old cultures grown at 20 °C in the dark in 90-mm plates on CA, V8A and potato–dextrose agar (PDA; HiMedia, Mumbai, India) according to patterns observed previously [9,31,61].

For temperature–growth relationships, all five isolates of *S. medusiformis* were sub–cultured onto 90 mm V8A plates and incubated for 24 h at 20 °C to stimulate onset of growth [118]. Then, three replicate plates per isolate were transferred to 5, 10, 15, 20, 22.5, 25, 27.5, 30, 32.5 and 35 °C. Radial growth was recorded after 4–10 days, before colonies reached the margin of the Petri dishes, along two lines intersecting the centre of the inoculum at right angles, and the mean growth rates (mm/d) were calculated. To determine the lethal temperature, plates showing no growth at 27.5, 30, 32.5 or 35 °C were returned to 20 °C.

## 3. Results

### 3.1. Phylogeny

**Figure 1:**
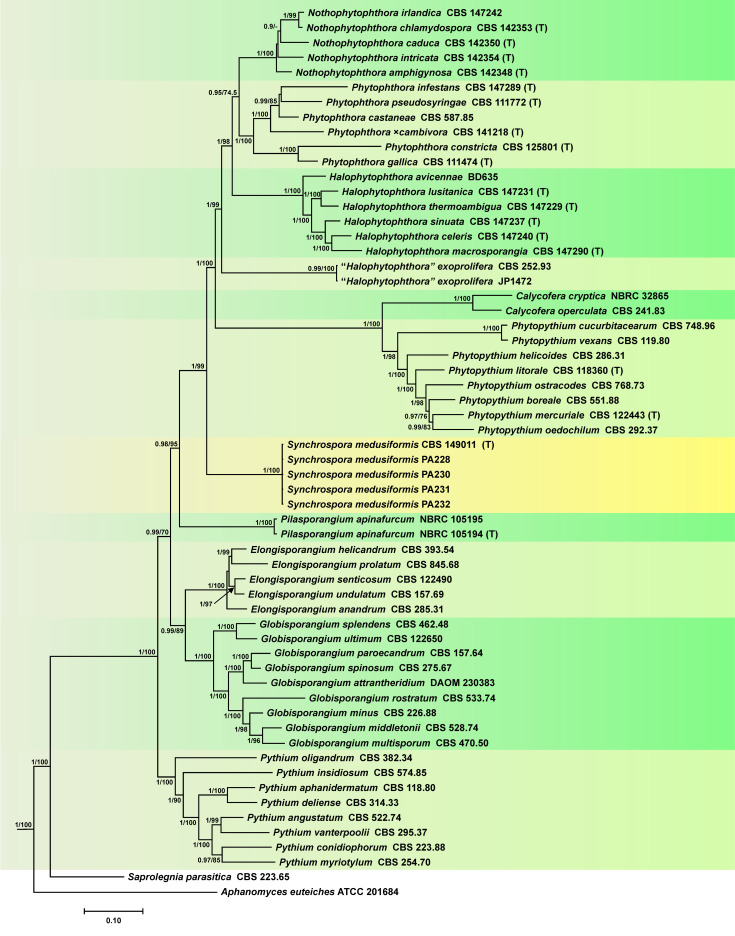
Fifty percent majority rule consensus phylogram derived from Bayesian phylogenetic analysis of a concatenated five–loci (LSU, ITS, *βtub*, *cox*1, cox2) dataset of *Synchrospora* gen. nov. and representative species from other genera of the Peronosporaceae and Pythiaceae. Bayesian posterior probabilities and maximum likelihood bootstrap values (in %) are indicated but not shown below 0.9 and 70%, respectively. *Saprolegnia parasitica* and *Aphanomyces euteiches* were used as outgroup taxa. Scale bar indicates 0.1 expected changes per site per branch.

Both the BI and the ML analyses of the 5–partition (LSU–ITS–*βtub*–*cox1*–*cox2*) dataset (4954 characters) produced phylogenetic trees with full support for both the deeper and end nodes and almost identical topology. The Bayesian tree is presented here with both Bayesian Posterior Probability values and Maximum Likelihood bootstrap values included (Figure 1, Dryad Dataset, https://doi.org/10.5061/dryad.p2ngf1vvt). The five known Peronosporaceae genera, *Calycofera*, *Phytopythium*, *Halophytophthora*, *Nothophytophthora* and *Phytophthora*, were well differentiated with full support, as were the Pythiaceae genera *Elongisporangium*, *Globisporangium*, *Pilasporangium* and *Pythium* (Figure 1). The mostly terrestrial soil-, air- and waterborne genera *Phytophthora* and *Nothophytophthora* constituted sister genera with the predominantly marine genus *Halophytophthora* residing in a basal position to them. “*Halophytophthora*” *exoprolifera* belonged to an undescribed distinct genus basal to the *Halophytophthora*–*Nothophytophthora*–*Phytophthora* cluster, while the cluster comprising the sister genera *Calycofera* and *Phytopythium* resided in a basal position to the other known Peronosporaceae genera, confirming recently published phylogenies [6,9,36]. The five isolates of the new species *Synchrospora medusiformis* formed a fully supported distinct clade that resided in a basal position to the cluster comprising the described Peronosporaceae genera, suggesting *Synchrospora* as the basal genus of the Peronosporaceae.

Across a 5-partition (LSU–ITS–βtub–cox1–cox2) alignment (4209 characters), S. medusiformis showed pairwise differences from its closest relative Pilasporangium apinafurcum at 614 positions, equivalent to a genetic distance of 14.6%. With 204 polymorphisms and 103 indels across a 788 bp alignment, differences were considerably higher (39.0%) in the ITS region with its non–coding parts than in the coding genes LSU (1260 bp; 86 polymorphic sites; 6.8% difference); βtub (918 bp; 99 polymorphisms and 6 indels; 11.4%); cox1 (680 bp; 64 polymorphisms; 9.4%); and cox2 (563 bp; 52 polymorphisms; 9.2%).

### 3.2. Taxonomy

Synchrospora T. Jung, Y. Balci, K. Broders and M. Horta Jung, gen. nov. MycoBank MB 847829.

Etymology. Name refers to the synchronous production of numerous sporangia from one sporangiophore apex.

Type species. *Synchrospora medusiformis*.

In the only known species, *Synchrospora medusiformis*, sporangiophores are usually unbranched or infrequently have a short lateral branch, showing determinate growth multifurcating at the end in a subdichotomous way, forming a stunted, candelabra-like apex from which multiple (8 to >100) long arch- or hook-like curved pedicels grow and form the sporangia in a synchronous way, resulting in a multi-sporangia structure with a medusa-like appearance. Sporangia are narrow and elongated, mostly cylindrical to allantoid, usually with an asymmetric base and a papillate apex when mature, and caducous. After shedding, the pedicels usually become twisted. Sporangia germinate directly with multiple hyphae or indirectly by releasing 2–5 biflagellate zoospores through a very narrow exit pore without a discharge tube. No internal sporangial proliferation was observed. Very rarely, external proliferation of the sporangiophore occurs some distance from the apex. Chlamydospores are not formed. The breeding system is homothallic and, hence, predominantly inbreeding, forming smooth-walled oogonia, containing plerotic thick-walled oospores with a large lipid globule, and paragynous antheridia. Hyphae often show undulating growth. Phylogenetically, *Synchrospora* belongs to the Peronosporaceae within the Peronosporales.

**Figure 2:**
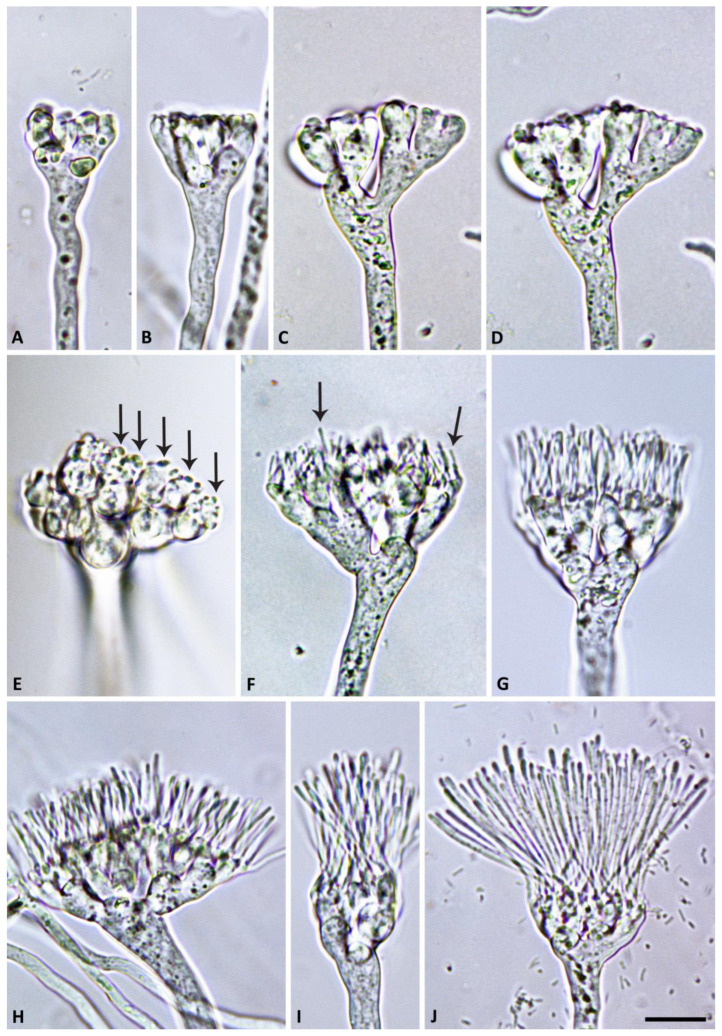
Morphological structures of *Synchrospora medusiformis* formed on V8 agar flooded with soil extract. (**A**–**D**) Candelabra-like branching of sporangiophore apices; (**E**,**F**) beginning growth of sporangial pedicels (arrows) from sporangiophore apices; (**G**–**J**) progressive growth of initially straight sporangial pedicels from sporangiophore apices. Scale bar: 15 µm.

**Figure 3:**
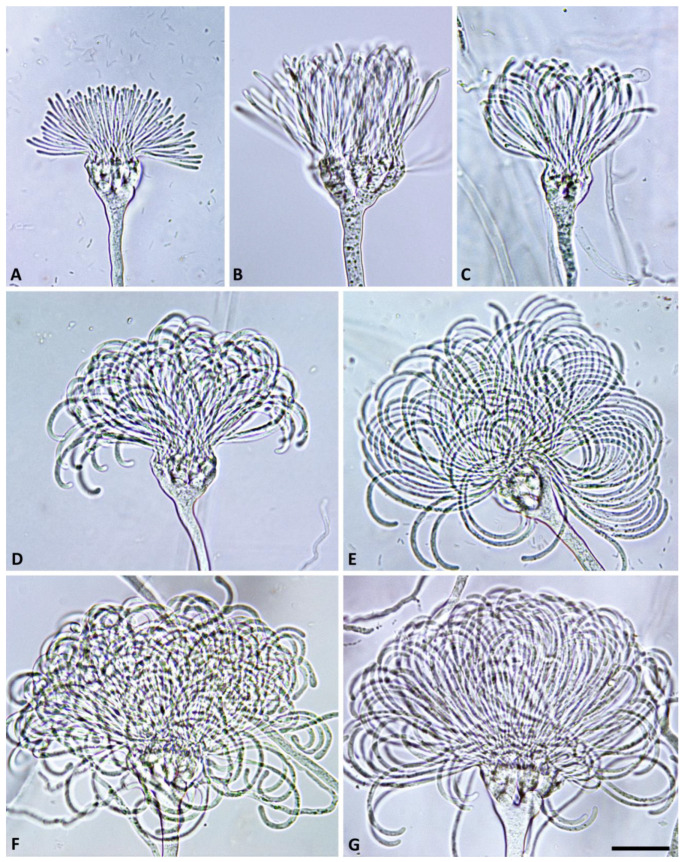
Morphological structures of *Synchrospora medusiformis* formed on V8 agar flooded with soil extract. (**A**,**B**) progressive growth of initially straight sporangial pedicels from sporangiophore apices; (**C**–**G**) progressive growth of increasingly curved, arch-like sporangial pedicels from sporangiophore apices. Scale bar: 25 µm.

**Figure 4:**
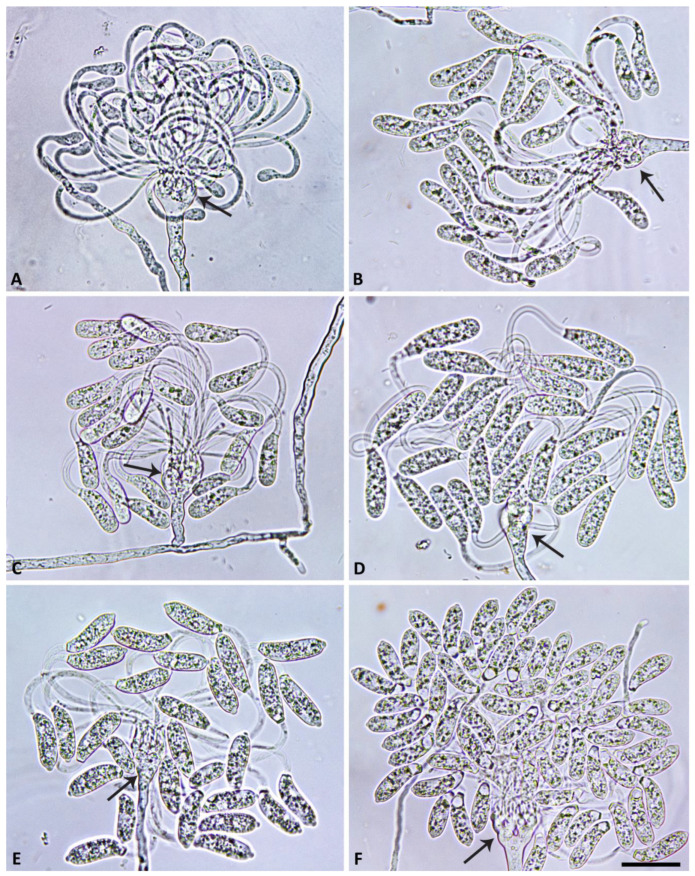
Morphological structures of *Synchrospora medusiformis* formed on V8 agar flooded with soil extract. (**A**–**F**) Medusa-like appearance of sporangiophore apices (arrows) with numerous long arch- or hook-like pedicels; (**A**–**D**) progressive formation of initially non-papillate sporangia at the ends of unbranched pedicels; (**E**,**F**) mature, papillate allantoid to tubular sporangia. Scale bar: 25 µm.

**Figure 5:**
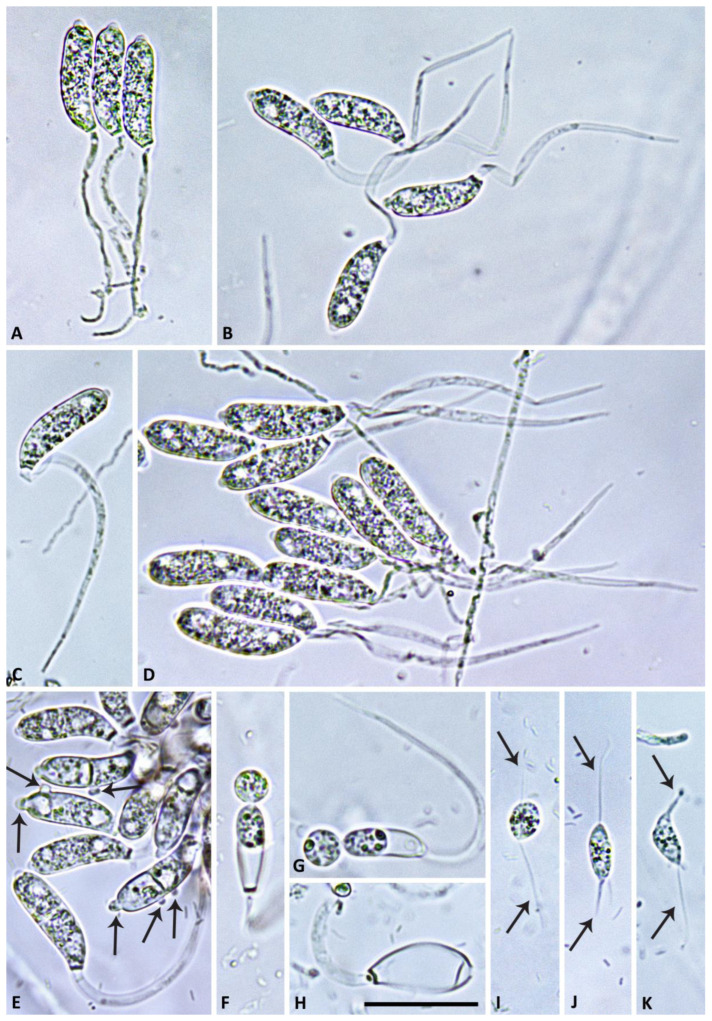
Morphological structures of *Synchrospora medusiformis* formed on V8 agar flooded with soil extract. (**A**–**E**) Mature, papillate, allantoid sporangia; (**A**–**D**) caducous with mostly curved or twisted long pedicels; (**E**) beginning direct germination (arrows); (**F**,**G**) allantoid sporangia releasing zoospores; (**H**) empty limoniform sporangium after zoospore release; (**I**–**K**) motile heterokont zoospores with each two flagella of unequal length (arrows). Scale bar: 25 µm.

*Synchrospora medusiformis* T. Jung, Y. Balci, K. Broders and I. Milenković, sp. nov.

(Figure 2, Figure 3, Figure 4 and Figure 5). MycoBank MB 847831.

Etymology: The name refers to the medusa-like appearance of the sporangiophore apex with multiple sporangia on long arch-like pedicels.

Holotype: Panama, Province Chiriqui, Volcano Barú, isolated from a naturally fallen leaf of an unidentified tree species in a tropical cloud forest at an altitude of 2393 m. Collected: K. Broders and Y. Balci, November 2019; CBS H-24948 (holotype, dried culture on V8A, Herbarium CBS–KNAW Fungal Biodiversity Centre), CBS 149011 = PA229 (ex-type culture). ITS, *βtub*, LSU, *cox1* and *cox2* sequences GenBank accession nos. OQ600177, OQ605385, OQ600184, OQ605396 and OQ605417, respectively.

Description: Sporangiophores were not observed in solid agar but were produced abundantly in non-sterile soil extract. They were usually unbranched or infrequently formed short lateral hyphae. Sporangiophores showed determinate growth and were always multifurcating at the end, forming a stunted, candelabra-like apex from which multiple (8 to >100) pedicels were growing simultaneously (Figure 2 and Figure 3). All sporangia at the end of the fully elongated pedicels from a sporangiophore apex were formed in a synchronous way, giving the mature multi-sporangia structure a medusa-like appearance (Figure 4). Sporangia were elongated, mostly cylindrical to allantoid (Figure 4 and Figure 5A–G) or very rarely limoniform (Figure 5h), usually with an asymmetric curved base and a papillate apex when mature (Figure 4E,F and Figure 5A–E). Sporangial dimensions of five isolates of *S. medusiformis* averaged 22.3 ± 2.6 × 7.2 ± 0.7 µm (overall range 15.8–31.6 × 5.5–9.2 µm), with a range of isolate means of 21.0–23.0 × 7.1–7.4 µm and a length/breadth ratio of 3.1 ± 0.4 (range of isolate means 2.96–3.21). Pedicels were arch-like or hook-like curved when still attached to the sporangiophore apex (Figure 3 and Figure 4). Pedicel length was 58.0 ± 9.6 µm (overall range 35.3–89.7 µm; range of isolate means 53.2–61.7 µm). All sporangia arising from a sporangiophore apex were caducous and shed more or less synchronously (Figure 5A–E). After shedding, the pedicels often became twisted (Figure 5A–E). Sporangia germinated directly with multiple hyphae (Figure 5E) or indirectly by releasing two to three (in rare cases up to five) zoospores through a very narrow exit pore (1.6 ± 0.2 µm) without a discharge tube (Figure 5F–H). Zoospores were heterokont biflagellate with one longer flagellum and one shorter flagellum and limoniform to reniform whilst motile (Figure 5I–K), becoming spherical (av. diam = 8.5 ± 1.1 µm) on encystment. No internal sporangial proliferation occurred. Very rarely, branching of the sporangiophore some distance from the apex was observed (Figure 4C).

**Figure 6:**
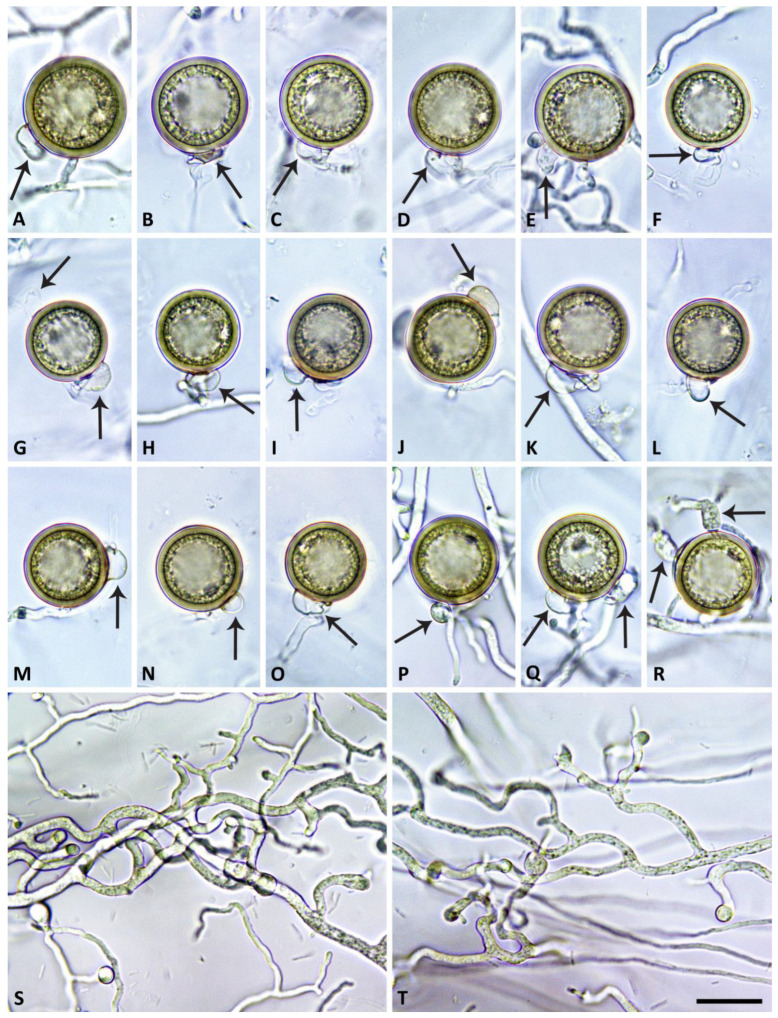
Morphological structures of *Synchrospora medusiformis* formed in solid V8 agar. (**A**–**R**) Mature, golden-brown, smooth–walled globose oogonia formed in single culture on very thin stalks, containing thick–walled fully plerotic oospores with large lipid globules (ooplasts), with one or sometimes two paragynous antheridia (arrows); (**S**,**T**) undulating hyphae without basal constrictions. Scale bar: 25 µm.

All five isolates of *S. medusiformis* were homothallic. Gametangia were produced in single culture in V8A within 10–14 d. Oogonia were borne terminally or laterally, had smooth walls and thin stalks and were globose with round, non-tapering bases (Figure 6A–R). The mean diameter of oogonia was 27.6 ± 2.9 µm (overall range 16.7–45.0 µm and range of isolate means 25.6–29.9 µm). They were exclusively plerotic, filling the oogonia completely and making a distinction between the oogonium and oospore walls in most cases impossible (Figure 6A–R). The oospores were globose with large lipid globules (=ooplasts), turning golden-brown during maturation (Figure 6A–R), and had a diameter of 26.0 ± 2.9 µm (overall range 15.1–38.8 µm), a wall diameter of 1.44 ± 0.19 µm (range 0.87–2.37 µm) and an oospore wall index of 0.30 ± 0.03. Oospore abortion was low (12.6% after 4 weeks). The antheridia were exclusively paragynous, club-shaped to subglobose, cylindrical or irregular (Figure 6A–R) and averaged 10.8 ± 2.4 × 6.1 ± 1.5 µm.

Hyphae often showed undulating growth (Figure 6S,T).

**Figure 7:**
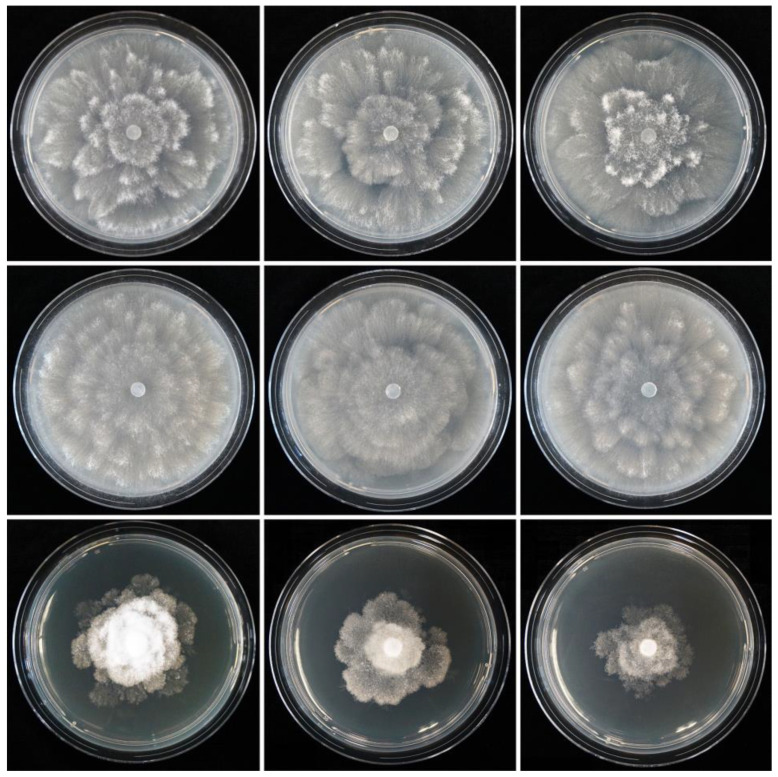
Colony morphology of *Synchrospora medusiformis* isolates CBS 149011, PA230 and PA231 (from left to right) after 7 d growth at 20 ºC on V8 agar, carrot agar, potato–dextrose agar and malt extract agar (from top to bottom).

**Figure 8:**
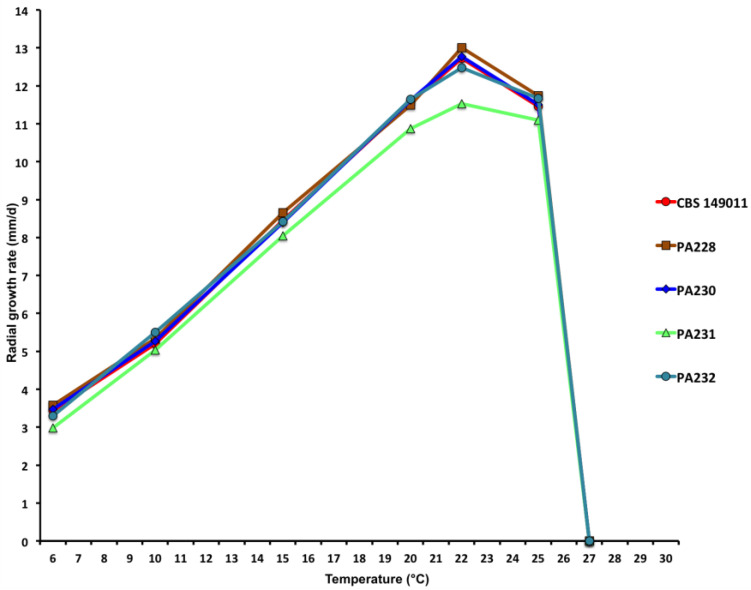
Mean radial growth rates of five isolates of *Synchrospora medusiformis* on V8 agar at different temperatures.

Colony morphology, growth rates and cardinal temperatures: Colonies of *S. medusiformis* on V8A and CA were largely submerged with limited aerial mycelium, with a petaloid pattern on V8A and a chrysanthemum pattern on CA. On PDA, colonies were densely felty with petaloid to stoloniferous patterns (Figure 7). Temperature–growth relations on V8A are shown in Figure 8. All five isolates included in the growth test had similar growth rates and cardinal temperatures. The maximum growth temperature was between 25 and 27.5 °C. The ex-type isolate (CBS 149011) did not resume growth when plates incubated for 7 d at 27.5 °C were transferred to 20 °C. The other four isolates did not resume growth when plates incubated for 7 d at 30 °C were transferred to 20 °C. The average radial growth rate on V8A at the optimum temperature of 22.5 °C was 12.5 ± 0.58 mm/d (isolate range 11.5–13.0 mm/d; Figure 8).

Other specimens examined (paratypes): Panama, Province Chiriqui, Volcán Barú, isolated from naturally fallen leaves of unidentified tree species in a tropical cloud forest at an altitude of 2393 m. Collected: K. Broders and Y. Balci, November 2019; PA228, PA230, PA231, PA232.

Notes: The only known species of the new genus *Synchrospora*, *S. medusiformis*, is differentiated from all other known oomycete species by its unique synchronous production of numerous (up to >100) caducous, long-pedicellate sporangia per multifurcating candelabra-like apex of sporangiophores with determinate growth enabling simultaneous aerial spread. In contrast, all aerial species of *Phytophthora* and *Nothophytophthora* produce sporangia individually on unbranched sporangiophores or consecutively on indeterminate sporangiophores, forming lax simple sympodia or dense compound sympodia [6,8,16,26,31,34,36]. Furthermore, the sporangia of *S. medusiformis* were, on average, smaller than in any known *Phytophthora* and *Nothophytophthora* species. Like *S. medusiformis*, all but two of the 20 known downy mildew (DM) genera also show determinate sporangiophore growth (*Viennotia* being the exception) and simultaneously ripening sporangia (*Sclerophthora* being the exception) [119]. Moreover, in several DM genera, the determinate sporangiophores also have dilated apices on which multiple sporangia or conidia are produced. These apices are saucer-shaped in *Bremia*, club-shaped in *Eraphthora*, cone- to club-shaped in *Basidiophora* and broad club-shaped to cylindrical in *Baobabopsis* [120,121,122,123]. However, none of the known DM genera form multifurcated candelabra-like sporangiophore apices. In addition, the pedicels of *S. medusiformis* are much longer than in any known DM species, and none of the known DM genera produce up to 100 or more sporangia per apex. Finally, the DMs are phylogenetically distant from *Synchrospora*, residing as two distinct clades within the paraphyletic genus *Phytophthora* [7,8,16], and are obligate biotrophic, nonculturable pathogens, whereas *S. medusiformis* grows well on various culture media.

## 4. Discussion

During a survey of oomycete diversity in natural forests of Central America, fast growing oomycete isolates were obtained alongside a diverse community of known and new *Phytophthora*, *Phytopythium* and *Pythium* species (Y. Balci, K. Broders and T. Jung, unpublished results), from naturally fallen tree leaves collected in a tropical cloud forest near the peak of Volcano Barú in Panama. Phylogenetic analyses of a five-partition dataset of sequences from the nuclear ITS, LSU and *βtub* genes and the mitochondrial *cox1* and *cox2* genes placed them into a distinct, previously unknown species belonging to a new genus described here as *Synchrospora* gen. nov. Based on its distinct phylogenetic position and unique set of morphological and physiological characteristics, the novel taxon is described here as *S. medusiformis*.

The multigene phylogenetic analysis demonstrated that *Synchrospora* resides in a basal position to a large cluster comprising all known Peronosporaceae genera, i.e., *Calycofera*, *Halophytophthora*, *Nothophytophthora*, *Phytophthora* (including the DMs) and *Phytopythium* [1,2,4,6,8,9,124]. Due to the phylogenetic position and the sporangial caducity of *S. medusiformis*, a common characteristic in the Peronosporaceae genera *Nothophytophthora* and *Phytophthora* including the DMs that has never been observed in any of the known Pythiaceae genera, *Synchrospora* is assigned to the Peronosporaceae constituting the basal genus of the family.

Despite the high number of oomycete surveys performed during the previous two decades in both managed and natural ecosystems across most continents, there is only one GenBank entry matching *Synchrospora* (ITS accession no. KM265501, 100% identical to *S. medusiformis*), which came from isolate E14413A (designated as Fungal sp. E14413A), obtained from stem tissue of the shrub species *Croton alnifolius* (Euphorbiaceae) during a fungal endophyte survey in a tropical cloud forest of Ecuador. This finding and the fact that all isolates of *S. medusiformis* examined in the present study originate from naturally fallen tree leaves in a remote tropical cloud forest near the peak of Volcano Barú in Panama suggest that this species is a neotropical canopy dweller in permanently humid cloud forests with a highly specialised aerial lifestyle as a leaf and bark pathogen.

Functionally, the synchronous production and ripening of up to more than 100 caducous sporangia per candelabra-like sporangiophore apex in *S. medusiformis* resembles 19 of the 20 DM genera [4,119,120,123,125,126,127], allowing simultaneous aerial spread with high inoculum pressure. Another similarity between *Synchrospora* and the DMs is the small size (and hence weight) of the sporangia increasing their aerial dispersibility, whereas the unusually long, curved and twisted pedicels most likely facilitate sporangial clustering and adherence to plant surfaces as recently suggested for aerial long-pedicellate *Phytophthora* species [16].

*Synchrospora medusiformis*, 75% of the eight described *Nothophytophthora* species, 26.7% of the 210 described *Phytophthora* species and all ca 900 DM species have caducous sporangia (or conidia) connected to an aerial or partially aerial lifestyle [4,6,13,14,16,31,34,36,123], whereas the other Peronosporaceae genera *Calycofera*, *Halophytophthora* and *Phytopythium* completely lack sporangial caducity [5,9,22,33]. Significant differences in sporangiophore growth and sporangial caducity between *Synchrospora* (determinate sporangiophores, synchronous production of up to >100 pedicellate caducous sporangia per candelabra-like sporangiophore apex); *Nothophytophthora* (indeterminate sporangiophores forming sympodia of non-pedicellate sporangia that mature non-synchronously; caducity by breaking off below a conspicuous opaque plug); *Phytophthora* (indeterminate sporangiophores forming sympodia of pedicellate sporangia that mature non-synchronously; caducity in airborne species); *Viennotia* (indeterminate sporangiophores, non-pedicellate caducous sporangia that mature non-synchronously); and the other 19 DM genera (determinate sporangiophores forming non-pedicellate or rarely pedicellate (cf. *Basdiophora*; [120]) caducous sporangia or conidia that mature synchronously) [3,4,6,16,26,34,36,119,123] suggest that sporangial caducity and an aerial or partially aerial lifestyle evolved independently in different Peronosporaceae genera in a convergent way.

During the past three decades, diversity of oomycete species and their ecological and pathogenic roles in soils and waterbodies of natural ecosystems have been studied extensively [29,37,38,39,40,43,44,45,46,49,50,51,52,53,54,57,60,65,67,68,70,73,75,76,78,80,82,83,84,85,87,88]. In contrast, there is only limited knowledge about the diversity and ecological roles of oomycetes in forest canopies, mainly gained from studies of often devastating diseases in temperate oceanic regions associated with (i) invasive aerial *Phytophthora* species like *P. ramorum* causing “Sudden Oak Death” in California and Oregon or “Sudden Larch Death” in the British Isles [43,48,62]; *P. kernoviae* causing aerial bark cankers on *Fagus sylvatica* in the UK [128]; *P. pinifolia* causing shoot and needle blight and defoliations of *Pinus radiata* in Chile [59]; *P. pluvialis* causing red needle cast of *P. radiata* in New Zealand [129] and branch and stem cankers and defoliations of *Tsuga heterophylla* in the UK [66]; or (ii) native aerial *Phytophthora* species like *P. pseudosyringae* causing bark cankers and dieback of exotic *Nothofagus* species in the UK [69]; *P. nemorosa* and *P. siskiyouensis* causing scattered bark cankers on *Notholithocarpus densiflorus* and other tree species in the Pacific Northwest [47]; and *P. ilicis* causing leaf, shoot and fruit blight on the native *Ilex aquifolium* in Sardinia, Italy [130]. Isolations from necrotic lesions of naturally fallen tree leaves indicate that in their centres of origin, *P. ramorum* (laurosilva forests in Vietnam and Japan); *P. kernoviae* and *P. pseudokernoviae* (Valdivian rainforests in Chile); and *P. celebensis*, *P. javanensis* and *P. multiglobulosa* (tropical rainforests in Indonesia) thrive in forest canopies as benign seasonal colonisers of senescent leaves [31,44,45,131,132]. Numerous leaf, shoot and fruit blights and canker diseases caused by aerial *Phytophthora* pathogens, including *P. botryosa*, *P. capsici*, *P. heterospora*, *P. meadii*, *P. megakarya*, *P. palmivora* and *P. tropicalis*, on tropical tree crops [8,26,133,134,135,136,137,138,139,140,141,142] and the findings of *S. medusiformis* on naturally fallen tree leaves and stem tissue in tropical cloud forests in Panama and Ecuador predict a rich community of aerial Phytophthoras and other oomycetes inhabiting tropical forest canopies. Extensive surveys in canopies of tropical lowland and montane forests using both isolation tests and metagenomic approaches from necrotic leaf, shoot, fruit and bark tissues, canopy-drip samples and spore traps are needed in both wet and dry seasons to unveil the diversity of tropical aerial oomycetes and their ecological roles and host associations.

The morphological and physiological attributes of P. parvispora provide insights into the ecology and survival strategy of this pathogen. Having high cardinal temperatures for growth >12, 27 and 37 °C, respectively, P. parvispora is well adapted to tropical and subtropical climates and greenhouse conditions, which is reflected by all known disease outbreaks.

## Figures and Tables

**Table 1:** Details of *Synchrospora* isolates used in the morphological and growth–temperature studies.

Species	Isolate Codes ^1^; Status ^2^	Host/Habitat	Location; Year; Collectors
*Synchrospora medusiformis*	CBS 149011 = PA229; T	Fallen leaf, tropical cloud forest	Panama, Volcano Baru; 2019; K.D. Broders andY. Balci
*S. medusiformis*	PA228	Fallen leaf, tropical cloud forest	Panama, Volcano Baru; 2019; K.D. Broders and Y. Balci
*S. medusiformis*	PA230	Fallen leaf, tropical cloud forest	Panama, Volcano Baru; 2019; K.D. Broders and Y. Balci
*S. medusiformis*	PA231	Fallen leaf, tropical cloud forest	Panama, Volcano Baru; 2019; K.D. Broders and Y. Balci
*S. medusiformis*	PA232	Fallen leaf, tropical cloud forest	Panama, Volcano Baru; 2019; K.D. Broders and Y. Balci

^1^ Abbreviations of isolates and culture collections: CBS = CBS collection at the Westerdijk Fungal Biodiversity Institute, Utrecht, Netherlands; PA: Culture collection of Mendel University in Brno, Czech Republic. ^2^ T, ex-type strain.

## Data Availability

All sequences generated during this study are available from GenBank, and accession numbers are given in Table S1. All datasets and trees derived from BI and ML analyses are available from DRYAD (https://datadryad.org, accessed on 24 April 2023) (Dryad Dataset, https://doi.org/10.5061/dryad.p2ngf1vvt).

## Supplementary Materials

The following are available online at https://www.mdpi.com/article/10.3390/jof9050517/s1, Table S1: Details of isolates from *Synchrospora* and related oomycete genera considered in the phylogenetic studies.
