# Supplementary material for: Synchrospora gen. nov., a New Peronosporaceae Genus with Aerial Lifestyle from a Natural Cloud Forest in Panama
Source: J Fungi (Basel). 2023 Apr 27;9(5):517. doi: 10.3390/jof9050517 (PMC10218844; doi:10.3390/jof9050517)
Supplement: Supplementary file 1 [file jof-09-00517-s001.zip › Jung et al._Synchrospora_Table S1_27 April 2023.pdf]

Synchrospora gen. nov., a new Peronosporaceae genus with aerial lifestyle from a natural cloud forest in Panama

T. Jung [1,2*], Y. Balci [3], K.D. Broders [4,5], I. Milenković [1,6], J. Janoušek [1], T. Kudláček [1], B. Đorđević [1] and M. Horta Jung [1,2]

1 Phytophthora Research Centre, Faculty of Forestry and Wood Technology, Mendel University in Brno, 613 00 Brno, Czech Republic; thomas.jung@mendelu.cz (T.J.); ivan.milenkovic@mendelu.cz (I.M.); josef.janousek@mendelu.cz (J.J.); tomas.kudlacek@mendelu.cz (T.K.); biljana.dordevic@mendelu.cz (B.D.); marilia.jung@mendelu.cz (M.H.J.)
2 Phytophthora Research and Consultancy, 83131 Nußdorf, Germany; dr.t.jung@gmail.com (T.J.)
3 USDA- APHIS Plant Protection and Quarantine, 4700 River Road, Riverdale, Maryland, 20737 USA; yilmaz.balci@usda.gov
4 Smithsonian Tropical Research Institute, Apartado Panamá, Panama City 0843-03092, Panama
5 USDA, Agricultural Research Service, National Center for Agricultural Utilization Research, Mycotoxin Prevention and Applied Microbiology Research Unit, Peoria, IL 61604, USA; kirk.broders@usda.gov
6 Faculty of Forestry, University of Belgrade, Kneza Višeslava 1, 11030 Belgrade, Serbia

Table S1. Details of isolates from *Synchrospora* and related oomycete genera considered in the phylogenetic studies. GenBank numbers for sequences obtained in the present study are printed in italics.

**Species**	**Isolate codes [1]; status [2]**	**Origin**		**GenBank accession numbers**		
		**Host / habitat**	**Location; year; collector; reference [3]**	**ITS**	**LSU**<br>***βtub***	***cox1***<br>***cox2***
Aphanomyces euteiches	ATCC 201684 = pea isolate	Root of *Pisum sativum*	Denmark; Funen; A.B. Petersen; Gaulin *et al.* 2018	OMPI01000392 [3]	OMPI01000392 [4]<br>OMPI01000009 [4]	OMPI01000379 [4]<br>OMPI01000379 [4]
Calycofera cryptica	NBRC 32865; T	Yellow leaf of *Avicennia* sp.	Belize; 1996; n.a.; Bennett et al. 2017a	KY604972	KY604972<br>n.a.	KY604975<br>MF598483
C. operculata	ATCC 44952 = CBS 241.83 = IMI 249911 = NBRC 32629; T	Decaying leaf of *Avicennia marina*	Australia, Moreton Bay; 1980; K.G. Pegg & J.L. Alcorn; Bennett *et al.* 2017	KJ128038	JX115217<br>n.a.	KF853238<br>MF598482
Elongisporangium anandrum	CBS 285.31; A/T	*Rheum rhaponticum*	USA; C. Drechsler; Robideau *et al.* 2011	HQ643435	HQ665185<br>JAADXR010000031 [4]	HQ708482<br>AB362328
E. helicandrum	CBS 393.54; A	*Rumex acetosella*	USA; n.a.; C. Drechsler; Lévesque & de Cock 2004	HQ643548	HQ665225<br>KJ595453	HQ708592<br>AB362329
E. prolatum	CBS 845.68; T	*Rhododendron* sp.	USA, Georgia; n.a.; W.A. Campbell; Lévesque & de Cock 2004	HQ643754	HQ665303<br>JAADVW010000542 [4]	HQ708795<br>AB362330
E. senticosum	CBS122490	Soil, deciduous broadleaf forest	Japan, Takayama; 2009; Senda & Kageyama; Nguyen *et al.* 2022	HQ643773	HQ665093<br>JAADVQ010000246 [4]	HQ708814<br>AB362317
E. undulatum	CBS 157.69 = IMI 323158	Soil under *Pinus* sp.	USA, Alabama; 1968; W.A. Campbell; Robideau *et al.* 2011	HQ643946	HQ665134<br>JAADVF010000028 [4]	HQ708987<br>KJ595348
Globisporangium attrantheridium	DAOM 230383			HQ643477	HQ665308<br>AB512819	HQ708524<br>AB512886
G. middletonii	CBS 528.74	Soil	The Netherlands; n.a.	AY598640	AY598640<br>KJ595457	HQ708738<br>AB362318
G. minus	CBS 226.88	Soil	UK, Berkshire; n.a.; G. Clark; Lévesque & de Cock 2004	HQ643696	HQ665168<br>KJ595446	HQ708740<br>AB362320
G. multisporum	CBS 470.50; T	Soil	USA, Illinois; A.W. Poitras; Lévesque & de Cock 2004	AY598641	HQ665239<br>KJ595455	HQ708744<br>AB362319
G. paroecandrum	CBS 157.64 = BPIC 1297	Loamy nursery soil	South Australia, Adelaide; 1962; O. Vaartaja; Uzuhashi *et al.* 2010	AY598644	AY598644<br>JAASFU010000644 [4]	HQ708772<br>DQ071391
G. rostratum	CBS 533.74 = BR 649 = DAOM 229266	Soil	The Netherlands, Oostelijk Flevoland; 1971; A.J. van der Plaats-Niterink; Lévesque & de Cock 2004	AY598696	HQ665252<br>KJ595512	HQ708808<br>KJ595388
G. spinosum	CBS 275.67	Compost	The Netherlands, Baarn; n.a.; A.J. van der Plaats-Niterink; Lévesque & de Cock 2004	AY598701	HQ665181<br>JAADVN010000284 [4]	HQ708834<br>KJ595366

Species	Isolate codes [1]; status [2]	Origin		GenBank accession numbers		
		Host / habitat	Location; year; collector; reference [3]	ITS	LSU<br>*βtub*	*cox1*<br>*cox2*
G. splendens	CBS 462.48	n.a.	USA; n.a.; J.T. Middleton; Lévesque & de Cock 2004	HQ643795	AY598655<br>AB512852	HQ708836<br>AB512921
G. ultimum	CBS122650	n.a.	France; n.a.; B. Paul; n.a.	HQ643864	HQ665103<br>KJ639291	HQ708905<br>KJ639199
Halophytophthora avicennae	BD635	Baiting; Rio Séqua estuary, Ria Formosa	Portugal, Tavira; 2015; T. Jung; Maia *et al.* 2022	OK033633	OK033575<br>OK091253	OK091198<br>*OQ605397*
H. celeris	CBS 147240 = BD885; T	Baiting; tidal channel in salt marsh, Ria Formosa	Portugal, Santa Luzia; 2015; T. Jung; Maia *et al.* 2022	OK033645	OK033587<br>OK091266	OK091210<br>*OQ605398*
H. lusitanica	CBS 147231 = BD686; T	Baiting; tidal pond in salt marsh, Ria Formosa	Portugal, Almancil; 2015; T. Jung; Maia *et al.* 2022	OK033663	OK033605<br>OK091284	OK091228<br>*OQ605400*
H. macrosporangia	CBS 147290 = BD639; T	Baiting; tidal channel in salt marsh, Ria Formosa	Portugal, Santa Luzia; 2015; T. Jung; Maia *et al.* 2022	OK033664	OK033606<br>OK091285	OK091229<br>*OQ605401*
H. sinuata	CBS 147237 = BD656; T	Baiting; tidal pond in salt marsh, Ria Formosa	Portugal, Santa Luzia; 2015; T. Jung; Maia *et al.* 2022	OK033671	OK033613<br>OK091293	OK091236<br>*OQ605402*
H. thermoambigua	CBS 147229 = BD651; T	Baiting; tidal pond in salt marsh, Ria Formosa	Portugal, Santa Luzia; 2015; T. Jung; Maia *et al.* 2022	OK033680	OK033622<br>OK091300	OK091244<br>*OQ605403*
"Halophytophthora" exoprolifera"	ATCC 76607 = CBS 252.93 = AN-1065 = IFO 32420; PT	Fallen leaf of *Bruguiera gymnorrhyza*	Japan, Okinawa; 1988; A. Nakagiri; Ho *et al.* 1992	HQ643132	HQ665174<br>n.a.	HQ708205<br>n.a.
"Halophytophthora" exoprolifera"	JP1472	Fallen leaf in mangrove stand	Japan, Okinawa; 2018; T.Jung; this study	*OQ600172*	*OQ600178*<br>*OQ605379*	*OQ605386*<br>*OQ605399*
Nothophytophthora amphigynosa	CBS 142348 = BD268; T	Stream baiting in atlantic forest	Portugal, Sintra; 2015; T. Jung; Jung *et al.* 2017a	KY788382	OK047739<br>KY788515	*OQ605387*<br>*OQ605404*
N. caduca	CBS 142350 = CL328; T	Stream baiting in Valdivian rainforest	Chile, Valdivia; 2014; T. Jung; Jung *et al.* 2017a	KY788401	KY788470<br>KY788531	*OQ605388*<br>*OQ605405*
N. chlamydospora	CBS 142353 = CL316; T	Stream baiting in Valdivian rainforest	Chile, Valdivia; 2014; T. Jung; Jung *et al.* 2017a	KY788405	KY788450<br>KY788535	*OQ616982*<br>*OQ605406*
N. intricata	CBS 142354 = TJ275 = RK113-1s; T	Rhizosphere soil of *Aesculus hippocastanum*	Germany, Wiesbaden; 2011; T. Jung; Jung *et al.* 2017a	KY788413	OK047740<br>*OQ605380*	*OQ605389*<br>*OQ605407*
N. irlandica	CBS 147242 = PR13-109; T	Stream baiting	Ireland; 2014; R. O'Hanlon; O'Hanlon *et al.* 2021	MW364574	MW364589<br>MW367157	*OQ605390*<br>*OQ605408*
Phytophthora castaneae	ATCC 36818 = CBS 587.85 = IMI 325914 = WPC P15598	Soil	Taiwan; n.a.; H.S. Chang; Robideau *et al.* 2011	MH620122	KX251102<br>KX251098	MN883602<br>MH551178
P. constricta	CBS 125801 = CH 55C3 = TJ0306 = VHS 16130; T	Kwongan heathland	Australia, WA; 2006; VHS; Rea *et al.* 2011, Jung *et al.* 2022	ON000729	ON000635<br>OM975908	ON013795<br>*OQ605409*
P. infestans	CBS147289 = TJ1504 = T30-4; ET	Laboratory cross between *Solanum tuberosum* strains 80029 and 88133	The Netherlands; 1993; A. Drenth; Chen *et al.* 2022	MZ753914	*OQ600179*<br>MZ736454	MZ736428<br>*OQ605410*
P. gallica	CBS 111474 = CPHST BL35 = WPC P16826 = GAL1; T	Rhizosphere soil of riparian *Quercus robur*	France; 1998; T. Jung; Jung *et al.* 2022	MG865497	KX252594<br>KX252590	MH136893<br>HM534964
P. pseudosyringae	CBS 111772 = TJ1528 = WPC P10437 = PSEU6; T	Rhizosphere soil of *Q. robur*	Germany, Gerolzhofen; 1997; T. Jung; Chen *et al.* 2022	AY230190	KX250983<br>KX250979	*OQ605391*<br>*OQ605411*
P. ×cambivora	CBS141218 = IT 5-3 = TJ197; NT	Rhizosphere soil of *Quercus pubescens*	Italy, Sicily; 2013; T. Jung; Jung *et al.* 2017b	KU899179	OK033630<br>KU899255	MZ736422<br>*OQ605412*
Phytopythium boreale	CBS 551.88	Soil under *Brassica caulorapa*	China; n.a.; Y. Yang-nian; Robideau *et al.* 2011	HQ643372	HQ665261<br>EF408882	HQ708419<br>EF408876
Ph. cucurbitacearum	CBS 748.96 = IMI 333340	n.a.	Australia, Northern Territory; 1989; J. Duff; De Cock *et al.* 2015	HQ643381	AY598667<br>KJ595460	HQ708428<br>AB690680

Species	Isolate codes [1]; status [2]	Origin		GenBank accession numbers		
		Host / habitat	Location; year; collector; reference [3]	ITS	LSU<br>***βtub***	***cox1***<br>***cox2***
Ph. helicoides	CBS 286.31; A/T	*Phaseolus vulgaris*	USA; n.a.; C. Drechsler; Robideau *et al.* 2011	HQ643383	HQ665186<br>JAADYE010000001 [4]	HQ708430<br>DQ071377
Ph. litorale	CBS 118360; T	Soil of *Phragmites australis*	Germany, Lake Constance; 2003; J. Nechwatal; De Cock *et al.* 2015	HQ643386	HQ665082<br>JAADYB010001007 [4]	HQ708433<br>KJ595418
Ph. mercuriale	CBS 122443 = STE-U 6204; T	Rhizosphere of *Macadamia integrifolia*	South Africa, Limpopo Province; n.a.; W.J. Botha; De Cock *et al.* 2015	AB725882	KF853236<br>KJ595466	KF853239<br>AB690666
Ph. oedochilum	CBS 292.37; A	–	USA; n.a.; C. Drechsler; Robideau *et al.* 2011	HQ643392	JAADXX010000902 [4]<br>EF408883	JAADXX010000773 [4]<br>JAADXX010000773 [4]
Ph. ostracodes	CBS 768.73	Clay soil	Spain, Ibiza; 1972; A.J. van der Plaats-Niterink; Robideau *et al.* 2011	HQ643395	AY598663<br>EF408880	HQ708442<br>AB108013
Ph. vexans	CBS 119.80 = BR 484	Soil	Iran; 1985; D. Ershad; De Cock *et al.* 2015	HQ643400	HQ643400<br>JAADXV010000033 [4]	HQ708447<br>GU133518
Pilasporangium apinafurcum	NBRC 105194 = MAFF 241059 = UZ300 ;T	Uncultivated soil	Japan, Wakayama; n.a.; n.a.; Uzuhashi et al. 2010	AB458660	AB458651<br>BCKD01000092 [4]	MAFF 241059 CoxI [5]<br>AB458820
Pi. apinafurcum	NBRC 105195 = MAFF 241060 = UZ301	Uncultivated soil	Japan, Wakayama; n.a.; n.a.; Uzuhashi et al. 2010	AB458657	AB458652<br>BCKE01000081 [4]	MAFF 241060 CoxI [5]<br>AB458818
Pythium angustatum	CBS 522.74	Soil	The Netherlands, Oostelijk Flevoland; A.J. van der Plaats-Niterink; Lévesque & de Cock 2004	HQ643437	AY598623<br>JAADXQ010000849 [4]	HQ708484<br>KJ595387
Py. aphanidermatum	CBS 118.80	n.a.	n.a.; 1966; E. Laville; Lévesque & de Cock 2004	HQ643438	AY598622<br>JAADXP010001025 [4]	HQ708485<br>JAADXP010000623 [4]
Py. conidiophorum	CBS 223.88 = APCC 4005a	Soil	UK, Berkshire; n.a.; G. Clark; Lévesque & de Cock 2004	AY598629	HQ665166<br>KJ595486	HQ708555<br>KJ595361
Py. deliense	ATCC 12280 = CBS 314.33; A/T	Necrotic stem of *Nicotiana tabacum*	Indonesia, Sumatra; 1932; A. Meurs; Lévesque & de Cock 2004	HQ643522	HQ665204<br>KJ595497	HQ708568<br>KJ595372
Py. insidiosum	ATCC 58643 = CBS 574.85 = CDC B-4296; T	Horse	Costa Rica; before 1985; L. Mendoza; Robideau *et al.* 2011	HQ643570	HQ665273<br>KJ595515	HQ708614<br>AF196597
Py. myriotylum	CBS 254.70	*Arachis hypogaea*	Israel; n.a.; Z.R. Frank; Lévesque & de Cock 2004	HQ643701	AY598678<br>JAADWI010000209 [4]	HQ708745<br>KJ595365
Py. oligandrum	CBS 382.34	Root of *Viola sp.*	UK; n.a.; C.G.C. Chesters; Robideau *et al.* 2011	HQ643715	HQ665223<br>JAADWG010000248 [4]	HQ708759<br>KJ595381
Py. vanterpoolii	CBS 295.37; T	Root and stem base of *Triticum aestivum*	UK; 1936; T.C. Vanterpool; Robideau *et al.* 2011	HQ643952	HQ665193<br>JAADVD010000154 [4]	HQ708993<br>JAADVD010000820 [4]
Saprolegnia parasitica	CBS 223.65 = IMI 268364	Young *Esox lucius*	The Netherlands; 1965; A.J. van der Plaats-Niterink; n.a.	ADCG02001922 [4]	ADCG02001922 [4]<br>ADCG02000918 [4]	ADCG02004126 [4]<br>ADCG02004126 [4]
Synchrospora medusiformis	CBS 149011 = PA229; T	Fallen leaf, tropical cloud forest	Panama, Volcano Baru; 2019; K. Broders & Y. Balci; this study	*OQ600177*	*OQ600184*<br>*OQ605385*	*OQ605396*<br>*OQ605417*
S. medusiformis	PA228	Fallen leaf, tropical cloud forest	Panama, Volcano Baru; 2019; K. Broders & Y. Balci; this study	*OQ600173*	*OQ600180*<br>*OQ605381*	*OQ605392*<br>*OQ605413*
S. medusiformis	PA230	Fallen leaf, tropical cloud forest	Panama, Volcano Baru; 2019; K. Broders & Y. Balci; this study	*OQ600174*	*OQ600181*<br>*OQ605382*	*OQ605393*<br>*OQ605414*
S. medusiformis	PA231	Fallen leaf, tropical cloud forest	Panama, Volcano Baru; 2019; K. Broders & Y. Balci; this study	*OQ600175*	*OQ600182*<br>*OQ605383*	*OQ605394*<br>*OQ605415*
S. medusiformis	PA232	Fallen leaf, tropical cloud forest	Panama, Volcano Baru; 2019; K. Broders & Y. Balci; this study	*OQ600176*	*OQ600183*<br>*OQ605384*	*OQ605395*<br>*OQ605416*
Synchrospora sp. [6]	E14413A	Stem, *Croton alnifolius* in tropical cloud forest	Ecuador, Mindo; n.a.; n.a.; n.a.	KM265501	n.a.	n.a.

n.a. = not available.

[1] Abbreviations of isolates and culture collections: ATCC = American Type Culture Collection, Manassas, USA; BD, CL and TJ: Dr Thomas Jung's personal culture collection, housed at Mendel University in Brno, Czech Republic and the University of Algarve, Faro, Portugal; CBS = CBS collection at the Westerdijk Fungal Biodiversity Institute, Utrecht, Netherlands; CH: Chuanxue Hong laboratory at Virginia Polytechnic Institute and State University, Virginia Beach, VA, USA; CPHST BL: USDA-APHIS-PPQ-Center for Plant Health, Science & Technology-Beltsville Laboratory, Beltsville, MD, USA; IMI: CABI Biosciences, UK; JP, PA: Culture collection of Mendel University in Brno, Czech Republic; DAOM = Canadian National Mycological Herbarium, Agriculture and Agri-Food Canada, Ottawa, Canada; IFO = Institute for Fermentation, Osaka, Japan; MAFF: Research Center of Genetic Resources, NARO, Tsukuba, Japan; NBRC: NITE Biological Resource Center, Tokyo, Japan; VHS: Vegetation Health Service Collection, Department of Environment and Conservation, Perth, Australia; WPC = World Phytophthora Collection, University of California Riverside, USA; other isolate names and numbers are as given by the collectors and on GenBank, respectively.
[2] T, ex-type strain; ET, ex-epitype strain; NT, ex-neotype strain; PT, ex-paratype strain; A, authentic strain, identified by the author of the species; A/T, authentic strain, probably used for original description.
[3] References:

Bennett, R.M.; de Cock, A.W.A.M.; Lévesque, A.; Thines. M. *Calycofera* gen. nov., an estuarine sister taxon to *Phytopythium*, Peronosporaceae. *Mycol. Prog.* **2017**, *16*, 947–954.

Chen, Q., Bakhshi, M., Balci, Y., Broders, K.D., Cheewangkoon, R., Chen, S.F., Fan, X.L., Gramaje, D., Halleen, F., Horta Jung, M., *et al.* (2022). Genera of phytopathogenic fungi: GOPHY 4. *Stud. Mycol.* **2022**, *101*, 417–564.

de Cock, A.W.A.M.; Lodhi, A.M.; Rintoul, T.L.; Bala, K.; Robideau, G.P.; Abad, Z.G.; Coffey, M.D.; Shahzad, S.; Lévesque, C.A. *Phytopythium*: molecular phylogeny and systematics. *Persoonia* **2015**, *34*, 25–39.

Gaulin, E.; Pel, M.J.C.; Camborde, L.; San-Clemente, H.; Courbier, S.; Dupouy, M.-A.; Lengellé, J.; Veyssiere, M.; Le Ru, A., Grandjean, F.; *et. al.* Genomics analysis of Aphanomyces spp. identifies a new class of oomycete effector associated with host adaptation. *BMC Biology* **2018**, *16*, 43.

Ho, H.H.; Nakagiri, A. A new species of *Halophytophthora* from Atlantic and Pacific subtropical islands. *Mycologia* **1992**, *84*, 548–554.

Jung, T.; Scanu, B.; Bakonyi, J.; Seress, D.; Kovács, G.M.; Durán, A.; Sanfuentes von Stowasser, E.; Schena, L.; Mosca, S.; Thu, P.Q.; *et al. Nothophytophthora* gen. nov., a new sister genus of *Phytophthora* from natural and semi-natural ecosystems. *Persoonia* **2017a**, *39*, 143–174.

Jung, T., Horta Jung, M.; Scanu, B.; Seress, D.; Kovács, D.M.; Maia, C.; Pérez-Sierra, A.; Chang, T.-T.; Chandelier, A.; Heungens, A.; *et al*. Six new *Phytophthora* species from ITS Clade 7a including two sexually functional heterothallic hybrid species detected in natural ecosystems in Taiwan. *Persoonia* **2017b**, *38*, 100–135.

Jung, T.; Milenković, I.; Corcobado, T.; Májek, T.; Janoušek, J.; Kudláček, T.; Tomšovský, M.; Nagy, Z.; Durán, A.; Tarigan, M.; *et al.* Extensive morphological and behavioural diversity among fourteen new and seven described species in *Phytophthora* Clade 10 and its evolutionary implications. *Persoonia* **2022**, *49*, 1–57.

Lévesque, C.A.; de Cock, A.W.A.M. Molecular phylogeny and taxonomy of the genus *Pythium*. *Mycol. Res.* **2004**, *108*, 1363–1383.

Maia, C.; Horta Jung, M.; Carella, G.; Milenković, I.; Janoušek, J.; Tomšovský, M.; Mosca, S.; Schena, L.; Cravador, A.; Moricca, S.; *et al.* Eight new *Halophytophthora* species from marine and brackish-water ecosystems in Portugal and an updated phylogeny for the genus. *Persoonia* **2022**, *48*, 54–90.

Nguyen, H.D.T.; Dodge, A.; Dadej, K.; Rintoul, T.L.; Ponomareva, E.; Martin, F.N.; de Cock, A.W.A.M.; Lévesque, C.A.; Redhead, S.A.; Spies, C.F.J. Whole genome sequencing and phylogenomic analysis show support for the splitting of genus *Pythium*. *Mycologia* **2022**, *114*, 501–515.

O'Hanlon, R.; Destefanis, M.; Milenković, I.; Tomšovský, M.; Janoušek, J.; Bellgard, S.E.; Weir, B.S.; Kudláček, T.; Horta Jung, M.; Jung, T. Two new *Nothophytophthora* species from streams in Ireland and Northern Ireland: *Nothophytophthora irlandica* and *N. lirii* sp. nov. *PLoS ONE* **2021**, *16*, e0250527.

Rea, A.J.; Burgess, T.I.; Hardy, G.E.St.J.; Stukely, M.J.C.; Jung, T. Two novel and potentially endemic species of *Phytophthora* associated with episodic dieback of kwongan vegetation in the south–west of Western Australia. *Plant Pathol.* **2011**, *60*, 1055–1068.

Robideau, G.P.; de Cock, A.W.A.M.; Coffey, M.D.; Voglmayr, H.; Brouwer, H.; Bala, K.; Chitty, D.W.; Désaulniers, N.; Eggertson, Q.A.; Gachon, C.M.M.; *et al.* DNA barcoding of oomycetes with cytochrome c oxidase subunit I and internal transcribed spacer. *Mol. Ecol. Resour.* **2011**, *11*, 1002–1011.

Uzuhashi, S.; Tojo, M.; Kakishima, M. Phylogeny of the genus *Pythium* and description of new genera. *Mycoscience* **2010**, *51*, 337–365.

[4] Genome sequence sourced from the GenBank Whole-Genome Shotgun contigs.
[5] Sequences retrieved from the website "GeneBank Project, NARO" (https://www.gene.affrc.go.jp/databases-micro_search_en.php, accessed on 24 April 2023).
[6] Submitted to GenBank as Fungal sp. E14413A.
